# Use of polymyxin B with different administration methods in the critically ill patients with ventilation associated pneumonia: a single-center experience

**DOI:** 10.3389/fphar.2023.1222044

**Published:** 2023-09-01

**Authors:** Rupeng Shi, Yuanyuan Fu, Yujing Gan, Danying Wu, Suming Zhou, Min Huang

**Affiliations:** ^1^ Department of Geriatric ICU, The First Affiliated Hospital of Nanjing Medical University, Nanjing, China; ^2^ Department of Pharmacy, The First Affiliated Hospital of Nanjing Medical University, Nanjing, China

**Keywords:** intensive care unit, ventilation-associated pneumonia, extensively drug-resistant Gram-negative bacilli, polymyxin B, nebulized, nephrotoxicity

## Abstract

**Background:** Whether nebulized polymyxin B should be used as an adjunctive therapy or substitution strategy to intravenous polymyxin B for the treatment of ventilator-associated pneumonia (VAP) remains controversial. This study’s aim is to evaluate the efficacy and safety of different administration ways of polymyxin B in the treatment of ventilator-associated pneumonia caused by extensively drug-resistant Gram-negative bacteria(XDR-GNB).

**Methods:** This retrospective cohort study enrolled ventilator-associated pneumonia patients caused by XDR-GNB treated with polymyxin B in the intensive care unit. Patients were categorized by the administration methods as intravenous (IV) group, inhaled (IH) group, and the intravenous combined with inhaled (IV + IH) group. Microbiological outcome and clinical outcome were compared in each group. The side effects were also explored.

**Results:** A total of 111 patients were enrolled and there was no difference in demographic and clinical characteristics among the three groups. In terms of efficacy, clinical cure or improvement was achieved in 21 patients (55.3%) in the intravenous group, 19 patients (50%) in the IH group, and 20 patients (57.1%) in IV + IH group (*p* = 0.815). All three groups showed high success rates in microbiological eradication, as 29 patients with negative cultures after medication in inhaled group. Among all the patients who had negative bacterial cultures after polymyxin B, the inhaled group had significantly shorter clearance time than the intravenous group (*p* = 0.002), but with no significant difference in 28-day mortality. Compared with intravenous group, a trend towards a lower risk of acute kidney injury was observed in inhaled group (*p* = 0.025).

**Conclusion:** From the perspective of minimal systemic renal toxicity, nebulized polymyxin B as a substitution strategy to intravenous polymyxin B for the treatment of ventilator-associated pneumonia caused by XDR-GNB is feasible.

## 1 Introduction

Ventilator-associated pneumonia (VAP) is one of the most common infections in patients receiving invasive mechanical ventilation (Papazian et al., 2020). As an important part of hospital acquired pneumonia (HAP), the pathogenic microorganisms are mainly Gram-negative bacteria such as *Pseudomonas aeruginosa*, *Klebsiella pneumoniae* and *Acinetobacter* baumannii (Bailey and Kalil, 2015). Drug resistance caused by these bacteria has gradually increased over recent years (Gupta et al., 2017). According to the statistical data from China Antimicrobial Resistance Surveillance System (CARSS) and the China Antimicrobial Surveillance Network (CHINET) regarding bacterial resistance in VAP, the isolation rates of drug-resistant bacteria have shown varying degrees of increase (Hu et al., 2018). Some reviews and guidelines recommend a combination regimen containing polymyxin B, tigecycline, ceftazidime-avibatam for the treatment of extensively drug-resistant Gram-negative bacteria(XDR-GNB) with varied activity, but the optimal combination regimen is still unknown (Guan et al., 2016; Gales et al., 2019). Tigecycline was sensitive to Carbapenem-resistant Enterobacteriaceae(CRE) and Carbapenem-resistant *acinetobacter* baumannii (CRAB), but not to Carbapenem-resistant *pseudomonas aeruginosa*(CRPA). Moreover, the minimal inhibit concentration (MIC) of tigecycline against CRE and CRAB also increased year by year. Ceftazidime-avibactam showed good antibacterial activity only against non-metalase-producing CRE and some CRPA, but not against CRAB. Therefore, due to the lack of effective antibacterial treatment against these resistant pathogens, polymyxins which had previously faded away from people’s view because of their toxic effects, have been reemerged in current clinical treatment with high sensitivity against CRAB, CRE, CRPA (Dm, 2009). Polymyxins have a unique mechanism of action involving disruption of the outer membrane integrity of Gram-negative bacteria that in addition to providing rapid bactericidal activity may enhance the activity of other antibiotic classes (Tsuji et al., 2019).

Currently, there are two kinds of polymyxin used in clinical practice, polymyxin B and colistin methanesulfonate (CMS). CMS is an inactive prodrug while polymyxin B can be directly administered as an active antibiotic (Nation et al., 2014). After intravenous infusion, the low lung tissue penetration of polymyxin may affect its efficacy in the treatment (Rodvold et al., 2011). Previous studies have found that nebulized antibiotic therapy can effectively increase the concentration of polymyxin in lung tissue (Lu et al., 2010). While ESCMID’s position paper recommends avoiding the use of nebulized antibiotics in clinical practice due to limited evidence from current clinical trials, international consensus guidelines for the optimal use of the polymyxins committee believes that the potential benefits outweighs the risks. The role of nebulized polymyxin treatment as an adjunction therapy or substitution to intravenous polymyxin remains still controversial in the treatment guidelines for VAP (Kalil et al., 2016; Tsuji et al., 2019; Tamma et al., 2022).

At present, clinical trials and meta-analysis regarding the treatment of VAP with intravenous and nebulized therapy mainly focus on CMS, and there is still limited information on clinical trials related to polymyxin B. Therefore, we proposed the clinical hypothesis that nebulized polymyxin B is supposed to be better than CMS due to its pharmacokinetics profile. The aim of this study is to compare the efficacy and side effects of three different administration methods including intravenous polymyxin B, inhaled polymyxin B, and intravenous combined with inhaled polymyxin B in the treatment of VAP caused by extensively drug-resistant Gram-negative bacteria(XDR-GNB).

## 2 Methods

### 2.1 Study design

This retrospective cohort study was conducted in nine intensive care units of a teaching hospital affiliated with Nanjing Medical University from January 2020 to October 2022. All the ICU departments included have followed the standard operating procedures of nebulized antibiotics. A total of 111 eligible patients were included. Patients were categorized by the administration methods as intravenous (IV) group, inhaled (IH) group, and the intravenous combined with inhaled (IV + IH) group. The dosage and administration of polymyxin B were determined by the treating physicians. The loading dose of intravenous polymyxin B was 2.0–2.5 mg/kg, with an infusion time of 1 h. After 12–24 h, a maintenance dose of 2.5–3 mg/(kg. d) was given in two divided doses, and continued to be infused for more than 1 h. Inhalation with polymyxin B was done at the dose of 50 mg twice a day in a solution of 5 mL of distilled water through vibrating mesh nebulizer. The study was approved by the Medicine Institutional Review Board of Jiangsu Province Hospital(2021-SR-550). Inclusion criteria included: age over 18 years; VAP caused by XDR-GNB; receiving nebulized or intravenous polymyxin B therapy for more than 3 days; sputum, endotracheal aspirates or bronchoalveolar lavage fluid were collected for culture before and after treatment. Exclusion criteria: age less than 18 years old, medication time less than 3 days, sequential use of intravenous and nebulized therapy, inhalation of other antibiotics, and the presence of lung diseases such as cystic fibrosis. All patients who met the inclusion criteria were enrolled in the study at the time of their initial diagnosis of VAP.

### 2.2 Endpoint

The primary endpoint of this study was the clinical outcome and bacteriologic outcome of VAP after the treatment. ICU length of stay, ventilation free time in 90 days, 28-day mortality, 90-day mortality, VAP-related mortality rate, and side effects associated with polymyxin B were secondary endpoints.

### 2.3 Data collection

The following data were extracted from the electronic medical record system: age, gender, reason for ICU admission, sequential organ failure assessment (SOFA) scores, acute physiology and chronic health evaluation (APACHE II) scores, clinical pulmonary infection scores (CPIS), microbiological culture and susceptibility test results, administration and dosage of polymyxin B and course of treatment, history of antibiotic use within 30 days, complications, length of stay in the ICU, duration of mechanical ventilation, clinical outcomes, microbiological outcomes, and drug-related side effects, all-cause mortality during hospitalization, as well as 28-day and 90-day mortality.

### 2.4 Definition

VAP was defined as a lung infection in patients who have received invasive mechanical ventilation for at least 48 h and was a part of hospital-acquired pneumonia in the intensive care unit.

VAP was confirmed if pneumonia developed more than 48 h after endotracheal intubation and mechanical ventilation. The diagnosis of pneumonia was based on the appearance of new or progressive infiltrates on imaging, as well as at least two clinical findings suggestive of pneumonia, including: increased cough, increased purulent sputum, fever (≥38°C) or hypothermia (<35°C), and leukocytosis (white blood cell count ≥10*10^∧^9/L) or leukopenia (white blood cell count <4*10^∧^9/L) (American Thoracic Society and Infectious Diseases Society of America, 2005).

Microbiological diagnosis was defined as pathogens isolated from respiratory samples, including sputum, endotracheal aspirates, and bronchoalveolar lavage fluid (BALF). XDR-GNB refers to Gram-negative bacteria (GNB) that are extensively drug-resistant, meaning they have acquired resistance to at least one agent in all but two or fewer antimicrobial categories (Magiorakos et al., 2012). When the bacterial concentration from quantitative sputum culture is ≥ 10^∧^^7^ cfu/mL, the bacterial concentration from endotracheal aspirates is ≥ 10^∧^^5^ cfu/mL, the bacterial concentration from BALF is ≥ 10^∧^^4^ cfu/mL, it was defined as a positive bacterial culture (Rea-Neto et al., 2008). Microbiological success was defined by the eradication of pathogenic XDR-GNB on the follow-up respiratory samples (Betrosian et al., 2008). The microbiological clearance time is defined as the time when the pathogenic microorganisms in the respiratory tract become negative after treatment with polymyxin B.

Clinical responses are classified as cure (relief of signs or symptoms of pneumonia, improvement in chest imaging, and discontinuation of antibiotic therapy), improvement (significant improvement in signs and symptoms of pneumonia, improvement in chest imaging, but still requiring antibiotic treatment), and failure (no significant response to treatment, persistent or worsening of signs or symptoms of pneumonia, septic shock, or even death) (Kofteridis et al., 2010). The clinical response of patients to polymyxin B was evaluated after the completion of polymyxin B application. The clinical efficacy will be evaluated by two experienced treating physicians after the final dose of polymyxin B. The CPIS and oxygenation index were assessed before the use of polymyxin B, on the seventh day after use, and on the 14th day after use. Ventilator-free time (VFT) on day 90 is typically defined as the period of time during which a patient remains alive and does not require mechanical ventilation (MV) in ICU (Contentin et al., 2014).Sepsis and septic shock are defined according to international guidelines for management of sepsis and septic shock 2021 (Evans et al., 2021).

The probable side effects associated with polymyxin B include nephrotoxicity, neurotoxicity (the most severe of which include myasthenia gravis syndrome or respiratory arrest caused by paralysis of the respiratory muscles) (Falagas and Kasiakou, 2006), and skin pigmentation (Zavascki et al., 2016). Treatment with aerosolized polymyxin B also carries a risk of bronchospasm. Acute kidney injury (AKI) is defined as an increase in serum creatinine by 0.3 mg/dL within 48 h or by a 50% increase in serum creatinine within 7 days (Kellum et al., 2013). Chronic kidney disease (CKD) was GFR <60 mL/min per 1.73 m^2^ for >3 months (Levey et al., 2020).Renal function is assessed with serum creatinine variations according to the classification of risk, injury, failure, loss, and end-stage of kidney disease (RIFLE) criteria (Hartzell et al., 2009). In order to eliminate the influence of blood purification on serum creatinine levels, patients who had undergone continuous renal replacement therapy before polymyxin B medication were excluded from the nephrotoxicity assessment process.

### 2.5 Statistical analysis

SPSS 26.0 software was used for statistical analysis. Mean ± SD was used to describe normally distributed continuous variables, and ANOVA was used for analysis. The median (interquartile spacing [IQR]) was used to describe the non-normally distributed continuous variables, and the multi-independent sample Kruskal–Wallis test was used for analysis. Categorical variables were expressed as frequency or ratio and analyzed using the χ2 test. *p* < 0.05 was considered statistically significant. Kaplan-meier curves were used to assess the difference in 28-day cumulative survival among the IV group, IH group, and IV + IH group.

## 3 Results

### 3.1 Patients’ demographics and baseline clinical characteristics

**FIGURE 1:**
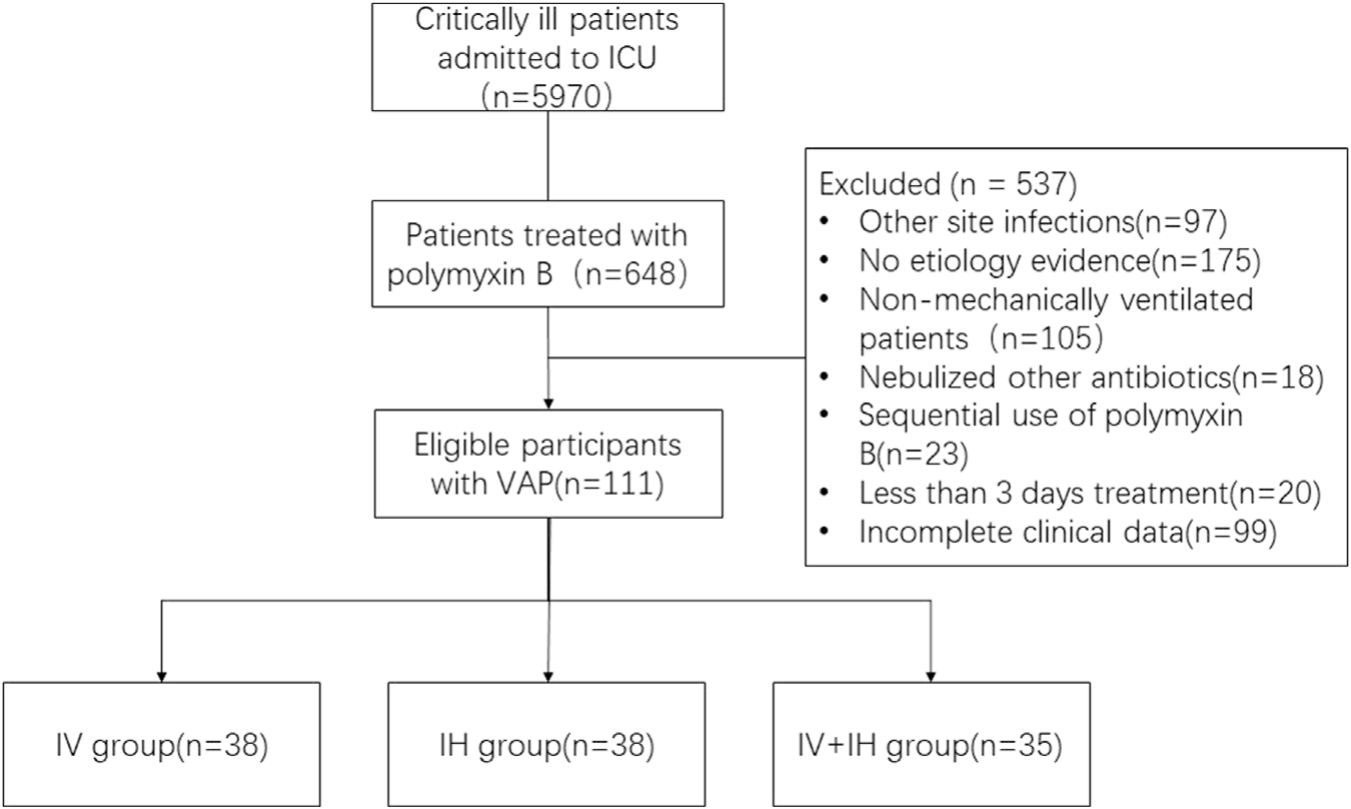
Study flow chart. VAP: ventilation associated pneumonia; IV: intravenous polymyxin B; IH: inhalation of polymyxin B; IV + IH: inhaled plus intravenous polymyxin B.

We enrolled 111 patients with confirmed VAP caused by XDR-GNB in Jiangsu Province Hospital from January 2020 to October 2022. Patients were recruited from various intensive care units (including three general intensive care units and six specialist intensive care units). As shown in Figure 1, among the enrolled patients, 38 patients received intravenous polymyxin B alone, 38 patients received nebulized polymyxin B, and 35 patients received combined treatment of intravenous and nebulized polymyxin B. There were 88 male patients (79.2%) and 23 female patients (20.8%). The mean age was (70 ± 17) years old. The median length of hospital stay was 33 days, and the median length of ICU stay was 31 days. Among the enrolled patients, 70.2% were admitted to the intensive care unit due to severe pneumonia and respiratory failure, while the rest were due to trauma or post operation.

All patients diagnosed with VAP were treated with polymyxin B combined with other intravenous antibiotics, of which carbapenems and β-lactam enzyme inhibitors (87.3%) were the most common simultaneously taken antibiotics. *Acinetobacter* baumannii (54.9%) was the most common pathogen, followed by *Pseudomonas aeruginosa* (26.1%) and *Klebsiella pneumoniae* (27.0%). There was no significant difference in microbial distribution among the groups (*p* = 0.227). In addition, due to the particularity of the intensive care unit environment, invasive operation and immuno-compromised state, 17.1% of the 111 patients enrolled in the study were found to have multiple bacterial infections in the respiratory pathogen detection before polymyxin B treatment.

The acute physiology and chronic health evaluation score at enrollment was 21 ± 5, the median sequential organ failure assessment score was 7(IQR: 5–10), and the clinical pulmonary infection score was 7(IQR: 5–8). There was no significant difference in APACHEⅡ score, SOFA score and CPIS score among the three groups. In IV + IH group, 37.1% of the patients were with lower oxygenation index (≤200) before medication, while 21.1% in the IV group and 23.7% in the IH group.

The mean length of hospital stay was 32 days (IQR: 24–54), with ICU stay time of 31 days (IQR: 23–48), and mechanical ventilation duration was 23 days (IQR: 15–40) (Table 1).

**TABLE 1:** Baseline demographic and patients’ clinical characteristics.

	IV group(n = 38)	IH group(n = 38)	IV + IH group(n = 35)	*p*-Value
Age, mean years ±SD	67 ± 17	73 ± 16	68 ± 17	0.213
Male, n (%)	30(78.9)	31(81.6)	27(77.1)	0.895
Cause of ICU admission, n (%)				
Medical	22(57.9)	28(73.7)	28(80)	0.100
Surgical	16(42.1)	10(26.3)	7(20)	
Comorbidities at admission, n (%)				
COPD	5(13.2)	5(13.2)	5(14.3)	0.987
Cardiovascular disease	16(42.1)	17(44.7)	18(51.4)	0.743
Neurological disease	19(50.0)	24(63.2)	23(65.7)	0.356
Diabetes mellitus	11(28.9)	6(15.8)	6(17.1)	0.330
Chronic renal failure	7(18.4)	5(13.2)	3(8.6)	0.539
Cancer	6(15.8)	9(23.7)	6(17.1)	0.685
causative agent, n (%)				0.227
XDR *Acinetobacter* baumannii	23(52.3)	20(51.3)	18(48.6)	
XDR *Pseudomonas aeruginosa*	6(13.6)	12(30.8)	11(29.7)	
XDR *Klebsiella pneumoniae*	15(34.1)	7(17.9)	8(21.6)	
Combination of pathogens	11(28.9)	5(13.2)	3(8.6)	0.050
Severity of disease				
APACHE II score, mean scores ±SD	20 ± 6	21 ± 4	21 ± 5	0.564
SOFA score, median (IQR)	8(5,11)	7(5,10)	6(5,8)	0.473
CPIS, mean scores ±SD	7 ± 2	6 ± 2	7 ± 2	0.481
Septic shock, n (%)	23(60.5)	26(68.4)	29(82.9)	0.109
Duration of polymyxin B therapy, median (IQR)	12(6,17)	11(8,17)	14(9,24)	0.033
Length of hospitalization, median (IQR)	42(26,58)	31(20,60)	31(21,50)	0.161
Length of ICU stay, median (IQR)	43.5(26,50)	30(20,48)	30(21,42)	0.409
Duration of mechanical ventilation, median (IQR)	27(17,41)	22(11,44)	21(17,33)	0.867
Concomitant Antibiotics,n (%)				
Beta-lactam	32(84.2)	36(94.7)	29(82.9)	0.247
Quinolones	4(10.5)	5(13.2)	4(11.4)	0.936
Tetracycline	12(31.6)	15(39.5)	16(45.7)	0.461
Glycopeptides	10(26.3)	2(5.3)	6(17.1)	0.044
Aminoglycosides	4(10.5)	2(5.3)	3(8.6)	0.761
Linezolid	3(7.9)	6(15.8)	2(5.7)	0.378
Antifungal drugs	14(36.8)	13(34.2)	11(31.4)	0.910

### 3.2 Therapeutic efficacy assessment

**FIGURE 2:**
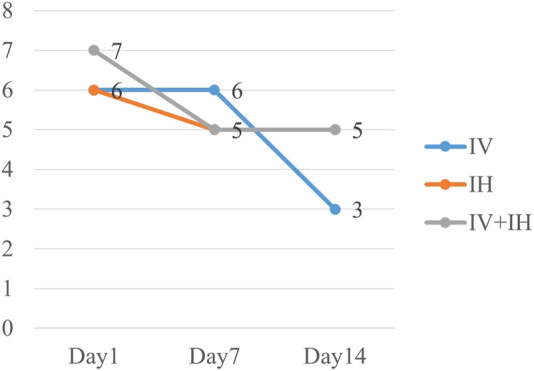
Evolution of CPIS score in each groups.

**FIGURE 3:**
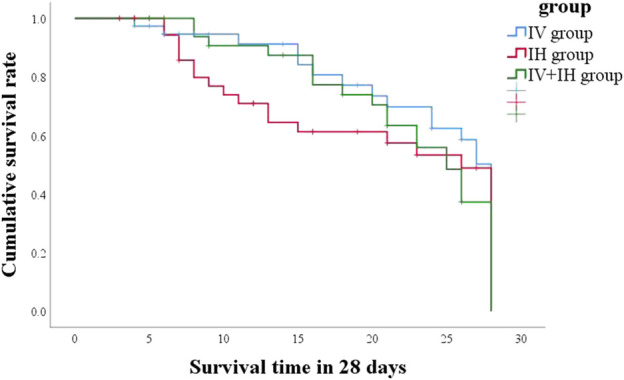
Survival analysis in each study groups.

In this study, 21 patients (55.3%) in IV group had good clinical outcome, compared with 50% in IH group and 57.1% in IV + IH group (*p* = 0.815) (Table 2). After treatment with polymyxin B, the clinical pulmonary infection scores of the three groups of patients showed a downward trend, but there were no significant differences between them (Figure 2). All three groups showed high success rates in microbiological eradication, 27 patients (77.1%) with negative cultures after medication in the combined treatment group. Among all the patients who achieved microbiological success, the IH group had significantly shorter clearance time than the IV group (*p* = 0.002), and similarly, the clearance time was significantly shorter in the IV + IH group than in the IV group(*p* = 0.049). In terms of prognosis, there were no statistically significant differences in all-cause mortality, VAP-related mortality, or 28-day mortality which showed that there is no significant difference in clinical efficacy. Kaplan-Meier analysis of all-cause mortality showed no significant difference in 28-day mortality among patients treated with different polymyxin B regimens (Figure 3).

**TABLE 2:** Clinical and bacteriological outcomes, and mortality in each treatment groups.

	IV group(n = 38)	IH group(n = 38)		IV + IH group(n = 35)	*p*-Value
Clinical outcomes, n (%)					
Clinical cure	6(15.8)	5(13.2)		4(11.4)	0.860
Clinical improvement	15(39.5)	14(36.8)		16(45.7)	0.730
Clinical failure	17(44.7)	19(50.0)		15(42.9)	0.815
Ventilation free time on day 90, median (IQR)	69(0,85)	59(0,84)		73(0,79)	0.867
In-hospital mortality, n (%)					
All cause	15(39.5)	15(39.5)		13(37.1)	0.973
28 days mortality	12(31.6)	12(31.6)		8(22.9)	0.641
90 days mortality	15 (39.5)	15(39.5)		13(37.1)	0.973
Bacteriological Eradication, n(%)	24(63.2)	29(76.3)		27(77.1)	0.359
Bacterial clearance time(days), median (IQR)	6(4,11)	3(3,6)		4(3,7)	0.002

**FIGURE 4:**
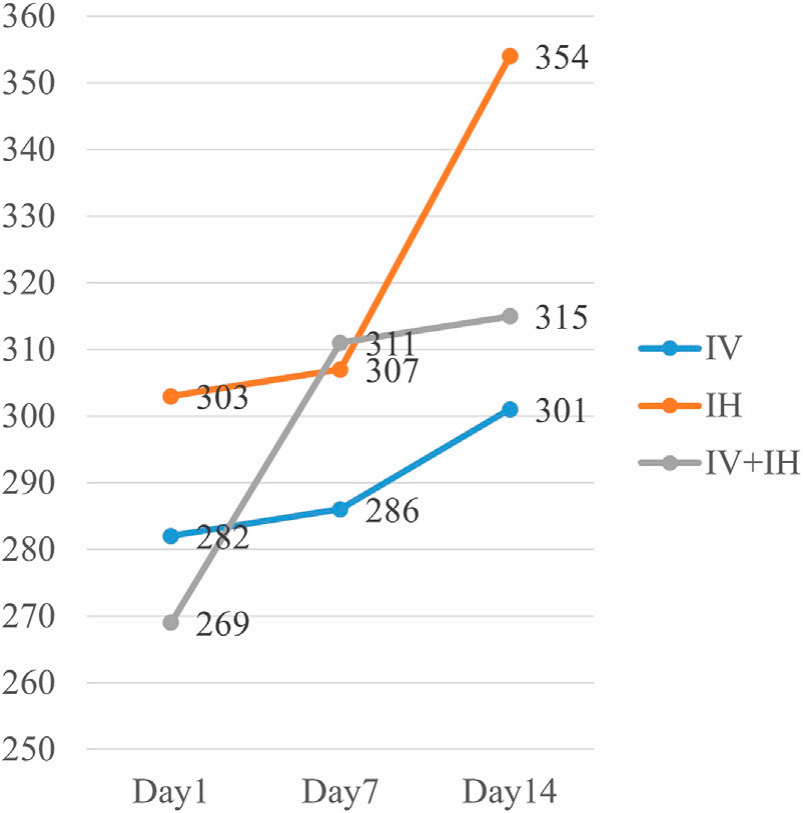
Evolution of PaO2/FiO2 ratio(P/F ratio) in each study groups.

In addition, the patients enrolled in this study were all assisted by ventilators before polymyxin B treatment, and more than 60% of the patients were in respiratory failure. After a 14-day course of treatment, the oxygenation index of the patients increased significantly, and the median oxygenation index of the IH group reached 354, but no significant difference was observed among the three groups (*p* = 0.378) (Figure 4).

### 3.3 Toxicity assessment

Before using polymyxin B, fifteen patients who received renal replacement therapy (RRT) were excluded in the assessment of nephrotoxicity. According to RIFLE criteria, the peak serum creatinine level during polymyxin B treatment is divided into different stages, including R(risk), I(injury), F(failure), L(loss), E(end-stage kidney diseases). Since the actual daily urine output of the patients could not be obtained very precisely, we used creatinine levels to determine the patients’ renal function. In our study, no patients met loss and end stage. As shown in Table 3, a total of 96 patients were enrolled in the nephrotoxicity assessment. There were no significant differences in baseline serum creatinine among the three groups of patients(*p* = 0.176). Compared to patients in IH group, patients in IV group are more likely to meet risk, injury, or failure (R, I, or F) criteria(25.0% VS. 62.3%) (*p* = 0.025). However, such differences were not found in the IV group and IV + IH group, as well as the IH group and IV + IH group.

**TABLE 3:** No. (%) of patients fulfilling criterion at peak creatinine level during polymyxin B treatment.

	IV group (n = 31)	IH group (n = 32)	IV + IH group (n = 33)	*p*-Value
Normal	12(38.7)	24(75.0)	20(60.6)	0.025
Risk	8(25.8)	3(9.4)	3(9.2)	
Injury	7(22.6)	4(12.5)	5(15.1)	
Failure	4(12.9)	1(3.1)	5(15.1)	

## 4 Discussion

This retrospective cohort study’s aim included critically ill patients with VAP caused by XDR-GNB is to evaluate the clinical efficacy and safety of polymyxin B in different administration method (IV, IH, IV + IH). Previous clinical studies mainly focused on CMS, and there were few clinical trials on the treatment of VAP with polymyxin B. In addition, previous researchers focused mainly on whether nebulized polymyxin B can be used as an adjunctive way to intravenous therapy rather than substitution strategy. This is the first study to compare three different administration methods of polymyxin B in the treatment of VAP caused by XDR-GNB. We found that the patients in IH group had shorter bacterial clearance time than IV group. This may be explained by the fact that inhalation can deliver polymyxin B directly to the site of infection. In addition, we also found that patients in the IH group had a lower risk of nephrotoxicity.

The epithelial lining fluid (ELF) is a common site of infection for most pathogenic bacteria, so the concentration of antibiotics in the ELF determines the effectiveness of treatment (Kiem and Schentag, 2008). Due to the existence of the alveolar-capillary barrier, also known as the blood-air barrier (BAB), some drugs like polymyxins with higher molecular weight or lower lipid solubility are difficult to penetrate through the tightly bound cellular barriers in the lungs. Therefore, their concentrations in the alveoli and lung tissue are relatively low. Some studies showed the concentration of colistin in ELF after intravenous administration was significantly lower than in plasma (Imberti et al., 2010). In a PK/PD study of mouse lung infection model caused by *Klebsiella* (Landersdorfer et al., 2018), the result showed a low penetration of polymyxin B into the lung parenchyma after systemic administration. Meanwhile, nebulized antibiotic allows the drug to reach a high concentration at the site of infection due to its direct action in respiratory tract while avoiding systemic toxicity and preventing the emergence of resistant strains (Ratjen et al., 2006; Lu et al., 2010; Palmer and Smaldone, 2014; Yapa et al., 2014). Therefore, nebulized antibiotics are increasingly being used by clinicians as a treatment option for respiratory infections in mechanically ventilated patients (Solé-Lleonart et al., 2016). The heated and humidified gas of the ventilator affects the size of drug particles, while the gas flow rate influences the percentage of particle impaction in the breathing circuit and proximal airways. These factors collectively limit the delivery of medication to the lungs (Miller et al., 2003; Ehrmann and Luyt, 2020). The variation in ventilator parameters among patients may have resulted in differences in drug concentrations in the lungs. Therefore, our next research direction is to directly measure the drug concentration in ELF, further evaluating the feasibility of nebulized inhalation of polymyxin B and its influencing factors.

But whether nebulized polymyxin B should be used as an adjunctive therapy or substitution strategy for the treatment of VAP remains controversial. According to the 2016 IDSA/AST guidelines for HAP/VAP and 2019 international consensus guidelines for the optimal use of the polymyxins, nebulized antibiotics can be considered as an adjunctive therapy for patients who are not responding to intravenous therapy alone (Kalil et al., 2016; Tsuji et al., 2019), while the ESCMID position paper recommended avoiding the routine use of nebulized antibiotics as an adjunctive or a substitution therapy in VAP patients with weak level of evidence and called for more future substitution RCTs (Rello et al., 2017).

In our study, inhalation was noninferior to intravenous therapy and concomitant therapy in the CPIS score, SOFA score, oxygenation index, ventilator-free days, and survival. In a previous retrospective cohort study from China, the results showed that the clinical cure rate and bacteriological clearance rate were not improved by the combination of intravenous and nebulized polymyxin B, but significantly favorable clinical outcome has been shown in the IH + IV group than in the IV group (Liu et al., 2022). But in our study there was no significantly favorable clinical outcome between IH, IH + IV and IV groups. Compared with the IV group, nebulized treatment shortened the bacterial clearance time in our study, which is consistent with the results of a previous prospective and randomized trial (Abdellatif et al., 2016).

In previous literature, the incidence of kidney injury in patients treated with polymyxins ranged from 0% to 60% (Kubin et al., 2012; Phe et al., 2014; Tuon et al., 2014; Rigatto et al., 2016; Zhang et al., 2018). The patient’s renal function, which is impaired after using polymyxins, usually improves with discontinuation of the drug, and permanent kidney damage is rarely seen (Ordooei Javan et al., 2015). Polymyxin B carries a certain risk of renal damage in its clinical application, which restricts its usage. Intravenous polymyxin B is more likely to cause renal impairment than nebulization. Our study also showed that patients in IV group are more likely to meet risk, injury, or failure (R, I, or F) criteria compared to patients in IH group. Previous clinical studies and guidelines have recommended that intravenous polymyxin B in combination with other antibiotics is rather than monotherapy (Vardakas et al., 2018; Perez et al., 2019). The combined use of vancomycin, aminoglycoside antibiotics, and other nephrotoxic drugs may promote the occurrence of AKI. Critically ill patients with severe infections are more susceptible to AKI due to multiple factors. Meraz’s research indicates that there is a risk of developing acute kidney injury after using colistin, and this risk increases the chances of developing chronic kidney disease within 6 months (Meraz-Muñoz et al., 2018). In order to study the occurrence of acute nephrotoxicity after the use of polymyxin B and the subsequent long-term renal function changes, additional studies about the mechanisms and the exploration of risk factors are required.

In addition to common renal toxicity, some literature also reported that treatment with aerosolized colistin may be complicated by cough, bronchoconstriction, and chest tightness (Antoniu and Cojocaru, 2012; Abdellatif et al., 2016). Among the patients included in this study, although the inhalation of polymyxin B was scheduled after conventional nebulizing agents (including corticosteroids, apophlegmatisant and bronchodilators), one patient was observed to have bronchospasm, which was relieved after nebulized acetylcysteine. Interestingly, this study found that patients in the IH group had the shortest VFT on day 90 among the three groups. Westerman et al. found that in some patients, a decrease in pulmonary function was observed after inhalation of colistin (Westerman et al., 2004). Colistin causes mast cell degeneration *in vitro*, which may be the cause of bronchoconstriction (Maddison et al., 1994). However, the exact mechanism has yet to be determined. The adverse respiratory reactions after nebulized inhalation still need more data for further study in the future. In addition, due to the fact that most patients are unconscious or sedated, it is difficult to detect neurotoxicity in the critically ill patient population. There has been no additional statistical analysis of neurological reactions in patients after medication.

The limitations of this study are as follows: 1) This study is a retrospective study, and further prospective randomized controlled trials with larger sample sizes are still needed in future to further verify the efficacy of different administration methods of polymyxin B; 2) The patients enrolled in this study were critically ill patients with an average age of 70 years, and the results may not be applicable to other general patients; 3) Only 25 patients had their clinical isolates tested for polymyxin B susceptibility (the cultured pathogens all showed susceptibility to polymyxin B); 4) As this study is retrospective, the respiratory parameters of patients using ventilators were different, and we did not assess factors like airway obstruction or lung consolidation. These factors might influence the effectiveness of inhaled polymyxin B; 5) Although current research suggests that the use of polymyxin B carries a risk of nephrotoxicity, the incidence and influencing factors vary greatly in various studies. Therefore, it is still necessary to focus on how to reduce the occurrence of AKI in the future.

## 5 Conclusion

This is the first retrospective cohort study to compare different administration methods of polymyxin B in the treatment of ventilator-associated pneumonia caused by XDR-GNB. We found no significant difference in clinical outcomes in each group, however, nebulized polymyxin B significantly shortened the bacterial clearance time. In addition, nebulized polymyxin B may alleviate nephrotoxicity. From the perspective of minimal systemic renal toxicity, nebulized polymyxin B as a substitute for intravenous administration of polymyxin B for the treatment of VAP caused by XDR-GNB may be feasible. Further large-scale randomized controlled trials are needed to evaluate the efficacy and safety of nebulized polymyxin B as substitution.

## Data Availability

The raw data supporting the conclusion of this article will be made available by the authors, without undue reservation.

## Abbreviations

VAP, ventilator-associated pneumonia; XDR-GNB, extensively drug-resistant Gram-negative bacteria; HAP, hospital acquired pneumonia; ICU, Intensive Care Unit; CRE, Carbapenem-resistant Enterobacteriaceae; CRAB, Carbapenem-resistant *acinetobacter* baumannii; CRPA, Carbapenem-resistant *pseudomonas aeruginosa*; CMS, colistin methanesulfonate; IV, intravenous polymyxin B; IH, inhaled polymyxin B; IV + IH, intravenous plus inhaled polymyxin B; SOFA, Sequential Organ Failure Assessment; APACHE II, Acute Physiology and Chronic Health Evaluation; CPIS, Clinical pulmonary infection score; VFT, Ventilator-free time; AKI, Acute kidney injury; GFR, Glomerular Filtration Rate; ELF, epithelial lining fluid; BAB, blood-air barrier.
